# The First Report on Agarwood Formation of *Aquilaria sinensis* (Lour.) Spreng Induced by *Fusarium equiseti*

**DOI:** 10.3390/plants14152272

**Published:** 2025-07-23

**Authors:** Libao Zhang, Jianglongze Yang, Ruiling Yuan, Dan Feng, Peng Chen

**Affiliations:** 1 Yunnan Academy of Forestry and Grassland, Kunming 650201, China; zlbao666@163.com (L.Z.); 15925560812@163.com (J.Y.); yuanruiling@yafg.ac.cn (R.Y.); fengdan@yafg.ac.cn (D.F.); 2 College of Forestry, Southwest Forestry University, Kunming 650224, China

**Keywords:** *Aquilaria sinensis*, *Fusarium equiseti*, agarwood, agarotetrol

## Abstract

*Aquilaria sinensis* (Lour.) Gilg, the exclusive botanical source of Chinese agarwood, holds significant medicinal value. This study investigated the agarwood-inducing potential of a *Fusarium* strain obtained through prior isolation work. Through integrated morphological characterization and molecular phylogenetic analysis, the strain was conclusively identified as *Fusarium equiseti*. GC-MS analysis revealed that fungal inoculation induced the synthesis of characteristic sesquiterpenes and aromatic compounds consistent with natural agarwood profiles. Quantitative determination demonstrated progressive accumulation of agarotetrol, a key quality marker, reaching 0.034%, 0.039%, and 0.038% at 2, 4, and 6 months post-inoculation, respectively—significantly exceeding levels from physical wounding (*p* < 0.05) and PDA control treatments. Histological examination showed characteristic yellow-brown oleoresin deposits concentrated in the inner phloem, mirroring the anatomical features of wild-type agarwood. Critical quality parameters measured in December-harvested samples included ethanol extractives (17.69%), chromone derivatives 2-[2-(4-methoxyphenyl) ethyl] chromone, and 2-(2-phenylethyl) chromone (2.13%), all meeting or surpassing the specifications outlined in the National Standard for Agarwood Classification (LY/T 3223-2020). These comprehensive findings establish *F. equiseti* as a promising microbial agent for sustainable agarwood production in *A. sinensis* plantations.

## 1. Introduction

*Aquilaria sinensis* (Lour.) Gilg, a member of the Thymelaeaceae family within the *Aquilaria* genus, is endemic to southern China’s subtropical regions, primarily inhabiting low-altitude evergreen broad-leaved forests in Guangdong, Hainan, Guangxi, Fujian, and Yunnan provinces. As the sole botanical origin of Chinese agarwood, this endemic species represents a pharmaceutically significant resource [1]. The formation of agarwood occurs as a defense response to biotic/abiotic stressors, where traumatic parenchyma cells synthesize oleoresin deposits—a complex mixture of sesquiterpenoids and phenylpropanoids—gradually developing into dark-brown to ebony aromatic resin [2]. Pharmacological studies have demonstrated its bioactivities, including anti-inflammatory (via modulation of NF-kB signaling pathways) [3], antitumor [4], and antidepressant [5]. The resin’s quality correlates with chromatic evolution [6], driven by progressive accumulation of sesquiterpenoids (e.g., agarospirol) and chromones (e.g., 6-hydroxy-2-(2-phenylethyl)chromone). Ecological surveys indicate that <7% of wild *A. sinensis* populations naturally develop commercially viable agarwood, exacerbated by unsustainable harvesting practices and habitat fragmentation. This ecological crisis has prompted China to classify it as a Grade II protected species. While artificial induction techniques (e.g., chemical stimulation, fungal inoculation) have reduced production timelines from decades to 12–24 months, persistent quality discrepancies in sesquiterpene diversity and chromone content compared to wild specimens highlight critical technological gaps.

Fungal induction has become a prominent method for agarwood production due to its operational efficiency and environmental compatibility. Current techniques primarily involve two approaches: (1) drilling holes in *A. sinensis* trunks to implant fungus-colonized media [7], or (2) infusing fungal suspensions into the xylem via perfusion systems [8]. Early studies by Tunstall (1929) demonstrated agarwood formation within 3–6 months using *Aspergillus* spp., *Fusarium* spp., and *Penicillium* spp., though the resin quality remained suboptimal [9]. Recent advancements identified specialized fungal strains with enhanced efficacy: Chen et al. isolated endophytic *Fusarium* spp. and *Lasiodiplodia* spp. from *A. sinensis*, confirming their superior induction capacity [10], while Subehan et al. achieved pharmacopeia-compliant agarwood within 12 months using *Fusarium* spp. [11]. These optimized strains enable standardized agarwood production within 1–2 years, delivering significantly improved yield while meeting quality benchmarks.

Through the study of the chemical constituents of agarwood samples at home and abroad, it was found that agarotetrol, 2-(2-phenylethyl) chromone, 2-[2-(4-methoxyphenyl) ethyl] chromone, and 2-(2-phenylethyl) chromone were the markers for the identification of agarwood [12,13]. The chemical constituents of agarwood mainly include sesquiterpenes, 2-(2-phenylethyl) chromones, fatty acid compounds, aromatic compounds, and other components [14]. The chemical constituents of agarwood obtained from different regions [15] or different tree species are quite different. Agarotetrol is a natural terpenoid compound, which is an active ingredient in agarwood medicinal materials. It can be dissolved in organic solvents such as methanol and has antibacterial, sedative, and anti-asthmatic effects. Many of the 2-(2-phenylethyl) chromone derivatives have potential pharmacological activities [16], and their content mainly reflects the difference in the quality of agarwood [17]. At present, the biosynthesis and regulation pathways of agarotetrol and 2-(2-phenylethyl) chromone are not clear enough. The question of how to induce the production of agarotetrol and 2-(2-phenylethyl) chromone in agarwood trees has become a key factor limiting the improvement of agarwood quality. In addition, the amount of ethanol extract is also one of the important indicators of the quality of agarwood. They may be related to the formation time and lipid content of agarwood [18]. The minimum content of ethanol extract in agarwood in national standards should be ≥10%. In the agarwood induced by fungi, the content of the above indicators is generally lower than that of wild agarwood. It is necessary to further screen the dominant strains with the function of inducing agarwood formation and improve the quality of the obtained agarwood.

This study aims to comprehensively evaluate the agarwood-inducing efficacy of a *Fusarium* strain (isolated from diseased *Macadamia integrifolia* in Yunnan, China) in *A. sinensis*, with specific focus on characterizing resin biosynthesis dynamics through the temporal quantification of volatile organic compounds and agarotetrol (2/4 months post-induction), validating the histochemical and physicochemical properties of matured agarwood against the National Standard LY/T 3223-2020 [19] via one-year induction trials, and benchmarking critical quality parameters—particularly ethanol extractives (≥17.69%), 2-[2-(4-methoxyphenyl)ethyl] chromone (≥2.13%), and 2-(2-phenylethyl) chromone—for pharmacological compliance. The integrated assessment seeks to establish mechanistic foundations for fungal induction while advancing standardized production protocols.

## 2. Materials and Methods

### 2.1. Materials

Plant material of *A. sinensis* was collected from cultivated plantations (10+ years old) in the Nabanhe River Watershed National Nature Reserve, Jinghong City, Xishuangbanna Prefecture, Yunnan Province, China (100°40′32″ E, 22°07′53″ N). Selected trees exhibited no prior resin formation, with trunk diameters exceeding 25 cm at a height of 1.3 m. The taxonomic identification was confirmed by Prof. Sima Yongkang (Yunnan Academy of Forestry and Grassland Sciences), with voucher specimens deposited in the institution’s herbarium (Accession No. YAF00011258). The experimental fungal strain *Fusarium* sp. YB-1, originally isolated from anthracnose-infected leaves of *Macadamia integrifolia* (Baoshan, Yunnan, 2018), was obtained from the Microbial Culture Collection of the Yunnan Academy of Forestry and Grassland Sciences. Reference agarwood samples (Chinese agarwood) (Chinese Pharmacopoeia Grade) were procured from Kunming Sheng’ai Traditional Chinese Medicine Museum.

### 2.2. Fungal Activation and Culture

Prior to activation, PDA medium was prepared by dissolving 23 g commercial PDA powder in 500 mL of distilled water within a 500 mL conical flask. After sealing with aluminum foil, the mixture was autoclaved at 121 °C (103 kPa) for 20 min, cooled to 50 °C, and aseptically dispensed into disposable plastic Petri dishes under laminar airflow. Active *Fusarium* YB-1 colonies exhibiting vigorous marginal growth were then aseptically transferred to fresh PDA plates using sterile bamboo tips in a laminar flow hood. Inoculated plates were sealed with parafilm and incubated at 25 °C under dark conditions until complete mycelial coverage was achieved (5–7 days).

### 2.3. Morphological and Molecular Identification

According to the classification system of Lester et al. [20], and according to the growth of strain YB-1 on PDA medium (25 °C constant temperature), the mycelial morphology, colony characteristics, spore morphology, and sporogenous cell structure were observed. The spore size was calculated using Image Pro Plus 6.0 software, and the preliminary identification was made. After identification, the above-mentioned strains were sent to the Chinese Typical Culture Center of Yunnan Agricultural University and Wuhan University for identification. The fungal species were identified using ITS (Internal Transcribed Spacer, ITS), LSU (Large Subunit of Ribosome, LSU), SSU (Sensory Specific Satiety Units, SSU), TUB (Tubby Bipartite Transcription Factor, TUB), TEF (Transcriptional Enhancer Factor, TEF), 28S (28S Ribosomal RNA, 28S), 18S (18S Ribosomal RNA, 18S) and seven fragments. The fungal gene sequence obtained using sequencing technology was compared with the reference gene sequence in the National Center for Biotechnology Information (NCBI) database using BLAST (v2.15.0+). The top 10 were screened for data analysis, and the representative strains were combined with the reference gene using MEGA11.0 software. The ML method was used to construct a phylogenetic tree [21].

### 2.4. Artificial Induction of A. sinensis

Healthy *A. sinensis* trees (≥10 years old, trunk diameter > 25 cm) were inoculated using a standardized drilling protocol: nine vertical rows of holes (1 cm diameter, 3–4 cm depth, 15 cm row spacing) were drilled 50 cm aboveground, with four radially distributed holes per row (7 cm spacing). Fungal inoculum (*Fusarium* YB-1 cultured on PDA) was aseptically inserted into pre-drilled holes using sterile bamboo skewers, sealed with parafilm, and replenished at 6-month intervals. Four treatments were implemented: (1) control (no drilling), (2) cold drill (drilling only), (3) PDA medium (sterile substrate), and (4) YB-1 inoculation. Resin formation was monitored through systematic sampling using a 15 cm sterile trephine at 2, 4, 6, and 12 months post-inoculation, collecting three radial core samples per tree across all xylem layers, with non-resinous control tissues sampled equivalently for comparative analysis.

### 2.5. Detection of Volatile Components in Processed Samples

Post-inoculation samples (2 months/4 months harvest) were air-dried, homogenized using a cryogenic grinder (Retsch MM400, 30 Hz, 2 min) with a 2 mm sieve, and composite aliquots (5 g) prepared for headspace solid-phase microextraction (HS-SPME) coupled with GC-MS analysis (Agilent 7890B/5977B MSD) via Yunnan Tongchuang Detection Technology Co., Ltd. (Kunming, China) Volatile extraction was performed by sealing 1.0 g of the sample in a 20 mL HS vial, equilibrating at 38 °C in a thermostatic water bath, and adsorbing compounds onto an SPME fiber for 30 min, followed by thermal desorption in the GC injection port for 2 min. Chromatographic separation employed a DB-5MS capillary column (30 m × 0.25 mm × 0.25 μm) with a temperature program from 50 °C to 300 °C at 5 °C/min. Compound identifications were confirmed through dual criteria: (1) mass spectral similarity >85% against the NIST/EPA/NIH 2020 database, and (2) experimental retention indices (RIs) calibrated with a C_8_–C_40_ n-alkane standard mixture. For compounds eluting before C_8_ (RI < 800), RIs were determined through extrapolation using the logarithmic retention time relationship between C_8_ and C_9_ alkanes according to the Kovats index system. All experimental RIs were required to be within ±15 units of literature values normalized to 5 °C/min using the correction formula: RI corr = RI ref − 4.5 × (5 − r ref), where r ref is the source ramp rate [22,23,24], with RI validation strictly following ISO 11024-2:1998 guidelines [25] for sesquiterpenes/chromones. Relative abundances were quantified via peak area normalization (signal/noise > 10:1), excluding compounds failing RI verification [26].

### 2.6. Determination of Agarotetrol Content

**Figure 1:**
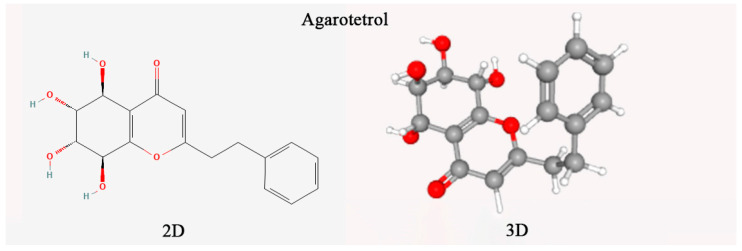
Two-dimensional/three-dimensional molecular representation of agarotetrol (5,6,7,8-Tetrahydroxy-2-(2-phenylethyl) chromone).

The agarotetrol content (chemical structure in Figure 1) was determined according to the 2020 edition of the Chinese Pharmacopoeia (Part I) [1]. A reference standard, 20 μg/mL of anhydrous ethanol (Lemeitian Pharmaceutical, CAS: 104834-43-1) was used. Agarwood samples were pulverized (<100 μm particle size) using a cryogenic mill. Precisely 0.2 g aliquots were ultrasonicated (Branson 5800, 250 W, 35 kHz) in 10 mL of ethanol (25 ± 2 °C, 60 min) with mass-loss compensation. Extracts were vacuum-filtered (Whatman No. 1) and further clarified through a 0.45 μm PVDF membrane to remove particulate matter. Chromatographic separation used an Agilent ZORBAX SB-C18 column (4.6 × 250 mm, 5 μm) with gradient elution: mobile phase A (acetonitrile) and B (0.1% formic acid) programmed as per Table 1 (0.7 mL/min flow rate, 30 °C). Detection at 252 nm (DAD) enabled quantification via external calibration (5-point curve, R^2^ = 0.9993). Method validation confirmed intra-day precision (RSD < 1.8%, n = 6) and recovery rates (98.2–102.4%). Data were analyzed using OpenLAB CDS ChemStation (v3.3).

### 2.7. Observation on Organizational Structure of Processed Samples

Sample preparation followed GB/T 29894-2013 wood identification standards [27]. Six-month specimens (Control, YB-1-treated, and pharmacopeial agarwood) were fixed in FAA solution (formalin:acetic acid:70% ethanol = 5:5:90 *v*/*v*) for 24 h, then sectioned into 1 cm^3^ cubes for triplanar microtomy (transverse/radial/tangential). Dehydration employed an ethanol–xylene gradient series: 50% ethanol (1% safranin O) → 70% → 80% → 95% → absolute ethanol (2 h/step), followed by xylene infiltration (ethanol–xylene ratios 4:1→3:2→2:3→1:4→pure xylene, 2 h/step). Paraffin embedding (HistoStar™) preceded sectioning on a rotary microtome (Leica RM2235, 5° blade angle) at 20 μm thickness. Selected sections were mounted on adhesive slides, deparaffinized with xylene, and counterstained with 1% fast green FCF in ethanol–xylene (1 min). Permanent mounts were prepared using neutral balsam (Sigma–Aldrich, St. Louis, MO, USA) under controlled humidity (40 ± 5% RH). Brightfield microscopy (Nikon Eclipse Ni-U) with DS-Fi3 camera captured resin deposition patterns at 40–400× magnification, analyzing oleoresin distribution within axial parenchyma and intervascular spaces.

### 2.8. Determination of the Content of Ethanol Extract of Agarwood

The agarwood samples obtained after 12 months were analyzed according to the provisions of the standard LY/T 2904-2017 [28], and 2 g of the crushed samples were weighed. The sample was placed in a 250 mL conical flask, and 100 mL of 95% ethanol was added to the pipette, sealed, weighed, and stood for 1 h. After that, the reflux condensation tube was connected, heated to boil, and kept for 1 h. After cooling, take off the conical bottle, close the plug, weigh again, use 95% ethanol to make up for the lost weight, shake well, and filter with filter paper. Next, 25 mL of the filtrate was taken with a pipette and placed in an evaporating dish that had been dried to a constant weight. After evaporation in a water bath, it was dried in an oven at 103 °C for 3 h and placed in a dryer. Cool for 30 min, weigh quickly, and calculate the content of ethanol extract in the test substance with the dry product.

### 2.9. Determination of Relative Content of 2-[2-(4-Methoxy)phenylethyl] Chromone and 2-(2-Phenylethyl) Chromone

**Figure 2:**
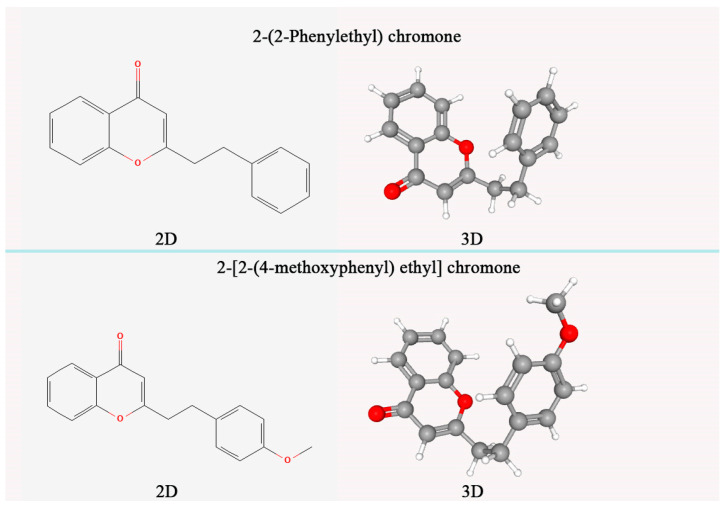
Comparative structures of key chromones in agarwood: 2-(2-Phenylethyl) chromone and 2-[2-(4-Methoxyphenyl) ethyl] chromone.

The preparation of agarwood reference sample and sample solution was carried out according to the provisions of LY/T 2904-2017 [28], in which the sample solution was determined by ethanol extract. Appropriate amounts of 2-[2-(4-methoxy) phenylethyl] chromone and 2-(2-phenylethyl) chromone standards (chemical structure in Figure 2) were accurately weighed (accurate to 0.0001 g), and 95% ethanol (analytically pure) was added to prepare a solution of 50 μg/mL. The filler was octadecylsilane-bonded silica gel (chromatographic column was Diamonsil C18, column length was 25 cm, inner diameter was 4.6 mm, particle size was 5 μm), mobile phase A was acetonitrile solution, mobile phase B was 0.1% formic acid solution, and gradient elution was performed according to the specified time (Table 2). The flow rate of the mobile phase was 1.2 mL/min. The column temperature was 31 °C. Detection wavelength at 252 nm.

## 3. Results

### 3.1. Fungal Identification Results

**Figure 3:**
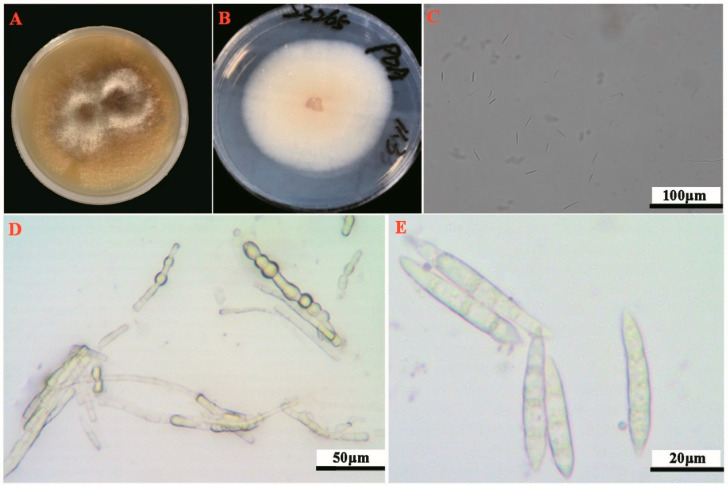
Morphological characteristics of strains and strains on PDA medium. Note: (**A**): strains used for activation culture, (**B**): colony morphology of the strain on PDA medium (positive), (**C**): macroconidia morphology, (**D**): optical microscope photo of sporogenous cells (200×), (**E**): optical microscope photo of macroconidia (400×).

The strain YB-1 was obtained using activation culture (Figure 3A) and grew well on PDA medium. The whole colony was milky white, the center was light yellow, slightly raised, the aerial mycelium was carpet-like, the back was light yellow, and there was no odor (Figure 3B). Large conidia gathered on the conidiophores, the mycelium was colorless, the wall was thin, and chlamydospores were not observed. The sporogenous cells are monophialides (Figure 3D), the conidia are sickle-shaped (Figure 3C), the cell wall is thin, and the cells at the top are elongated, usually divided into 3–4 parts (Figure 3E). The average size of the conidia is 104.89 μm^2^, the diameter is 13.71 μm, the length is 25.22 μm, and the width is 5.03 μm. According to the morphological comparison, the strain was preliminarily identified as *Fusarium* sp.

**Figure 4:**
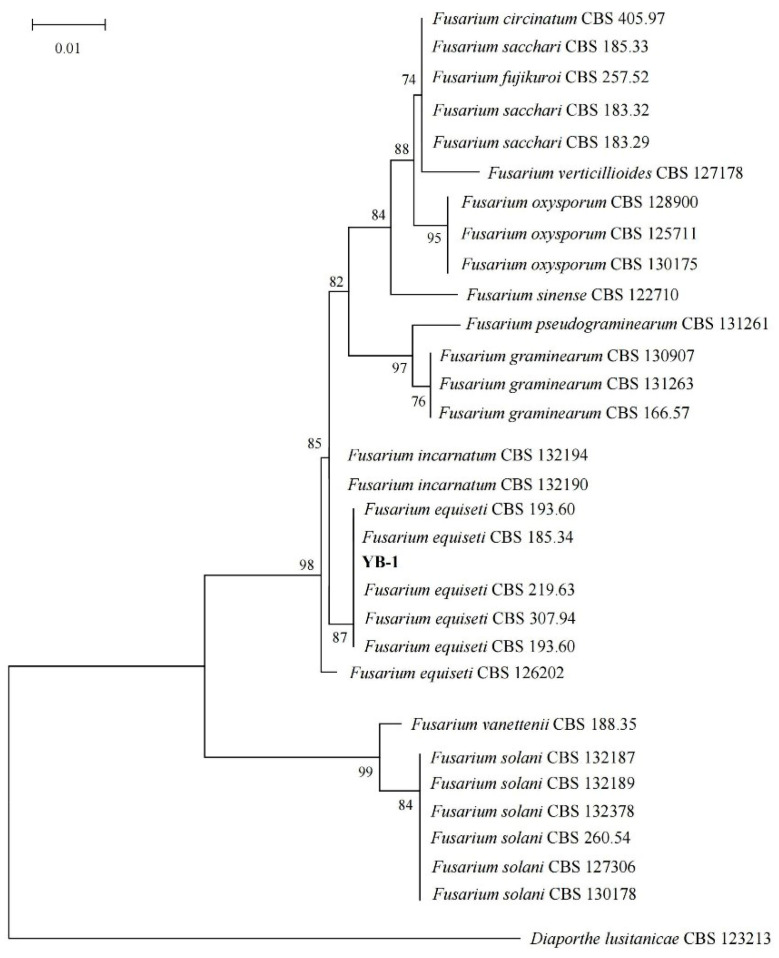
ML phylogenetic tree of *Fusarium equiseti* and its related species based on LSU and SSU fragments.

An analysis of the sequences obtained using gene sequencing revealed the following 28S sequencing alignment results: *Fusarium equiseti*, *Fusarium scirpi* sequence alignment consistency was 100%; ITS fragment alignment results: *Fusarium incarnatum*, *Fusarium equiseti* consistency of 100%; 18S alignment results: the consistency of *Fusarium equiseti* accounted for 99.88%; the results of LSU alignment showed that the identities of *Fusarium wereldwijsianum*, *Fusarium clavum*, *Stenocarpella maydis*, *Fusarium flagelliform*, *Fusarium incarnatum*, *Fusarium oxysporum*, *Fusarium lacertarum*, and *Fusarium irregulare* were 99.88%. SSU alignment results: the consistency of *Fusarium equiseti* was 100%; TEF alignment results: the consistency of *Fusarium equiseti* was 100%; TUB alignment results: the consistency of *Fusarium flagelliforme* and *Fusarium equiseti* was 100%. The phylogenetic tree (Figure 4) showed the closest relationship with *Fusarium equiseti*. The results showed that the phylogenetic tree was finally determined to be *Fusarium equiseti* by comparison results and multi-gene joint construction.

### 3.2. Analysis of Volatile Components of Agarwood Samples

GC-MS analysis confirmed (Table 3) that YB-1-treated *A. sinensis* wood generated core stress-response metabolites critical for agarwood formation (Table 4 and Table 5): β-Eudesmol (pharmacopoeia-specified marker) surged by 93% (1.98%→3.83%), acetic acid (wound-signaling molecule) escalated 530% (0.94%→5.93%) to promote sesquiterpene cyclization, β-Elemene (fungal stress biomarker) was consumed via >90% conversion after 2 months, and 2-pentylfuran (oxidative damage indicator) rose 263% (0.08%→0.29%) correlating with resin coloration. Concurrent secondary shifts mirrored natural resinogenesis: defense-activating hexanal increased by 247% (0.59%→2.05%), anaerobic metabolite ethanol surged 1126% (0.27%→3.31%), facilitating resin exudation, alkanes-like pentadecane decreased by 40% (3.02%→1.82%), confirming wound-response, and sesquiterpene byproduct alloaromadendrene increased by 21% (0.38%→0.46%). This coordinated transition—where sesquiterpenes and aromatics rose 122% and 40% (e.g., naphthalene +336%) while alkanes/short-chain acids declined 48%/62%—mechanistically validates YB-1′s efficacy in activating agarwood’s defense pathway, establishing its candidacy for pharmacological induction.

### 3.3. HPLC Fingerprint Characterization

**Figure 5:**
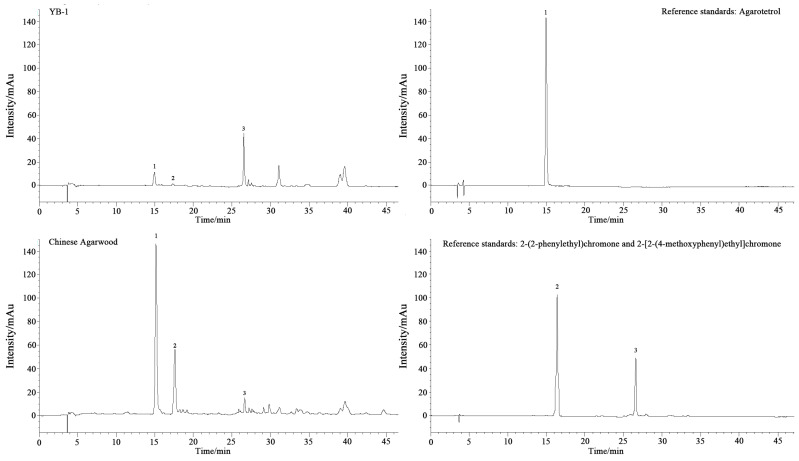
Comparative HPLC chromatograms of YB-1-induced agarwood, Chinese agarwood, and reference standards. Note: 1: Agarotetrol, 2: 2-(2-Phenylethyl) chromone, 3: 2-[2-(4-methoxyphenyl) ethyl] chromone.

In the chromatogram of agarwood treated with YB-1 for 6 months (Figure 5), the peak at 15.13 min (Peak 1) exhibited identical retention time to the agarotetrol reference standard (deviation < 0.2 min), confirming its identity as agarotetrol. Peaks 2 (17.21 min) and 3 (26.68 min) matched the reference standards for 2-(2-phenylethyl) chromone and 2-[2-(4-methoxyphenyl) ethyl] chromone, respectively. Compared with Chinese agarwood (which displayed multiple late-eluting peaks > 35 min, indicating enrichment of highly oxidized sesquiterpenes), the YB-1 sample showed a significantly simplified metabolic profile. The peak height of agarotetrol in YB-1 was only ~20% of that in the natural sample (based on relative peak area ratio), aligning with subsequent quantitative results where agarotetrol content in YB-1 (0.038%) was markedly lower than in natural agarwood (0.173%). Notably, the symmetry factor (Tailing Factor, *TF*) of Peak 1 in YB-1 (1.05) showed no significant difference (*p* > 0.05) from that of the natural sample (1.12), eliminating risks of quantitative bias from matrix interference.

### 3.4. Analysis of Agarotetrol Content

YB-1 significantly increased agarotetrol content within 2–4 months of treatment (0.034–0.039%), outperforming PDA (0.014–0.025%) and cold drill (0.002–0.006%) (*p* < 0.05). At 6 months, YB-1 (0.038%) and cold drill (0.037%) showed parity, while PDA declined to 0.004% (Table 6). Despite YB-1′s efficacy, its agarotetrol remained significantly lower than the medicinal benchmark (Chinese agarwood, 0.173%). We propose that YB-1 activity may decline over extended treatment (re-inoculation at 4–6 months is advised), necessitating optimization to approach natural agarwood’s pharmacological quality.

### 3.5. Microstructural Characteristics of Processed Samples

**Figure 6:**
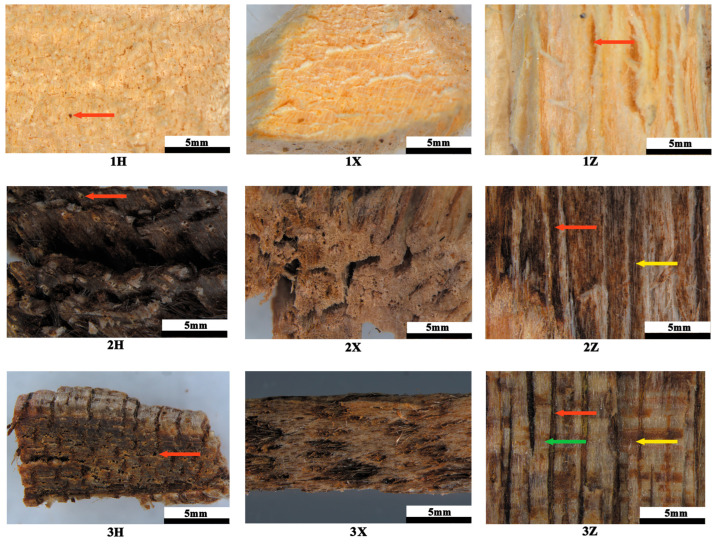
Macrostructure diagram. Note: 1: blank treatment, 2: YB-1 treatment, 3: Chinese medicine agarwood; H: transverse cutting, X: string cutting, Z: longitudinal cutting; red arrows: vessels, green arrows: wood rays, yellow arrows: oily substances.

Macroscopic analysis: *A. sinensis* wood exhibits a soft texture and is classified as diffuse-porous wood. The cross-section of the untreated (blank control) wood appeared yellowish-white and lacked visible oily substances (Figure 6). In contrast, all sections of wood treated with YB-1 and sections of traditional Chinese medicine agarwood displayed a yellowish-brown coloration. Notably, the intensity of this coloration in the transverse sections correlated positively with the abundance of oily substances; darker sections indicated a higher concentration of these substances. An examination of the longitudinal sections revealed fine, densely arranged wood rays, with yellow-brown oily substances filling the inter-ray spaces (Figure 6(2Z~3Z)). The tangential sections also exhibited oily substances of varying intensity. Within the transverse sections, vessel elements of *A. sinensis* were observed predominantly as solitary pores or in radial multiple tube holes. These vessels were numerous and relatively small in size (Figure 6(1H~3H)). The TCM agarwood sample exhibited a relatively soft texture, with a loose arrangement of vessel openings in its tangential section (Figure 6(3X)). It possessed a distinct medicinal aroma, characterized by notes of sweetness and a subtle alcoholic nuance. The surface presented a dark brown, natural appearance. Furthermore, its transverse section contained areas of uninfected sapwood (light-colored wood) and decayed wood (Figure 6(2H)).

**Figure 7:**
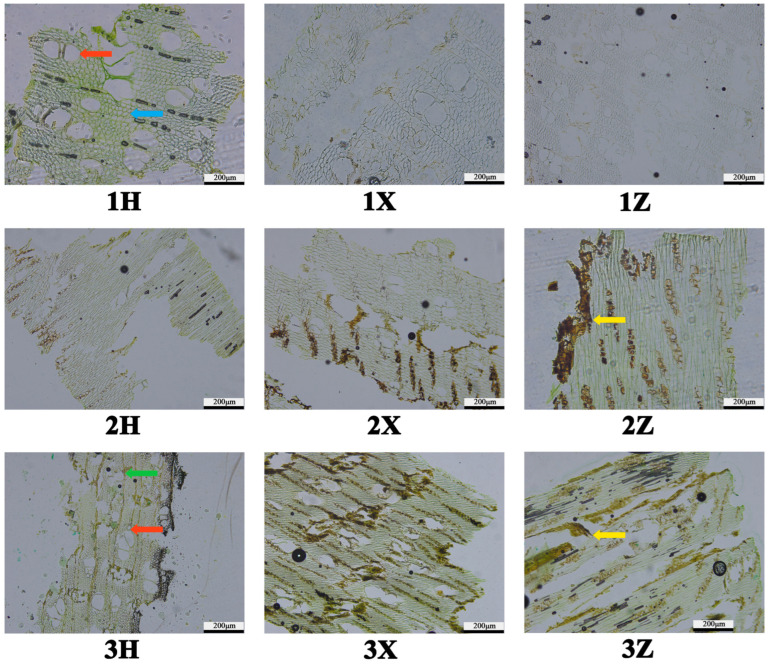
Microstructure diagram. Note: 1: blank treatment, 2: YB-1 treatment, 3: Chinese medicine agarwood, H: transverse cutting, X: string cutting, Z: longitudinal cutting red arrows: vessels, green arrows: wood rays, blue arrows: wood fibres, yellow arrows: oily substances.

Microscopic analysis: Growth rings were not distinctly discernible on the cross-sections of A. sinensis (Figure 7(1~3)). Axial parenchyma was inconspicuous, exhibiting a vasicentric arrangement. Vessel elements displayed diameters ranging from 45 μm to 98 μm and predominantly featured irregular outlines and multiple apertures per vessel element. Vessel walls were generally thin, averaging approximately 3 μm in thickness, and lacked tyloses or other occlusions. Wood fibers were polygonal in cross-section, slender, and arranged in parallel; most exhibited comparable shapes and sizes, with an average diameter of about 30 μm. Included phloem was distributed in island-like patterns between vessel multiples and clusters. Vessel lumina within the untreated (sapwood) sample remained devoid of oily substances, and the cellular structure was clearly defined without significant deformation; wood ray width appeared normal, showing no evidence of pronounced thickening, thinning, artificial scratches, or drilling artifacts (Figure 7(1)). In contrast, transverse sections of YB-1-treated wood and traditional Chinese medicine agarwood (TCM agarwood) exhibited substantial deposits of yellowish-brown oily substances, which accumulated predominantly within the included phloem. A positive correlation was observed between the intensity of sample coloration and the abundance of these oily deposits (Figure 7(2~3)).

### 3.6. Content Analysis of Ethanol Extract, 2-[2-(4-Methoxy)phenylethyl] Chromone and 2-(2-Phenylethyl) Chromone of Agarwood

After 12 months of induction of *A. sinensis*, the ethanol extract content (Table 7) of all agarwood treated with *Fusarium equiseti* YB-1 was significantly higher than that of cold drill treatment and treatment, reaching 17.69%. In addition, the sum of 2-[2-(4-methoxyphenyl) ethyl] chromone and 2-(2-phenylethyl) chromone reached 2.13%, which met the basic requirements of the national standard LY/T3223-2020 for agarwood grading. No 2-[2-(4-methoxyphenyl) ethyl] chromone and 2-(2-phenylethyl) chromone were produced under cold drilling and PDA treatments. This shows that after drilling (cold drilling treatment), the *A. sinensis* trees have a stress effect, enhance the secondary metabolism of *A. sinensis* trees, and produce agarwood substances; however, the effect and rate of forming agarwood are not ideal. 2-[2-(4-methoxyphenyl) ethyl] chromone and 2-(2-phenylethyl) chromone did not appear within 12 months, and the content of ethanol extract was relatively low. The addition of *F. equiseti* YB-1 greatly accelerated this process. After 12 months of induction, agarwood that met the national standard could be produced, which proved that *F. equiseti* YB-1 was the dominant strain to promote agarwood formation.

## 4. Discussion

In nature, fungi are easy to obtain and culture. Many fungi can continuously produce chemical components highly similar to natural agarwood when inducing agarwood plants, which has the advantages of high efficiency, environmental protection, and low cost. However, while the diversity of fungal species is immense, it is well-established that certain fungi can act as pathogens, potentially causing decay or death in plants. This inherent risk underscores the critical importance of screening for effective fungi capable of inducing agarwood formation without causing significant harm. At present, some fungal species have been proven to promote agarwood formation, such as *Aspergillus* spp., *Botryodyplodia* spp., *Fusarium* spp., and *Penicillium* spp. [7,10,29,30]. Tabata et al. [31] found that five different species of *Fusarium sp*. were inoculated into *A. sinensis*, and after a period of observation, agarwood was produced. Huang Qiuwei et al. [32] isolated 11 strains of endophytic fungi from *A. sinensis*, of which the better fungi were *Fusarium oxysporum*, *Fusarium fujikuroi*, *Fusarium proliferatum*, *Fusarium solani*, *Lasiodiplodia pseudotheobromae*, and *Lasiodiplodia theobromae*. *Botryosphaeria* sp. was isolated from *A. sinensis* by Zhang Yao et al. [29,30]. In vitro experiments showed that *Botryosphaeria* sp. could induce *A. sinensis* branches to produce agarwood in vitro. In this study, we focused specifically on identifying and evaluating beneficial fungi for agarwood induction. *A. sinensis* induced by *Fusarium equiseti* not only promoted agarwood formation, but also the content of ethanol extract, 2-[2-(4-methoxyphenylethyl)] chromone, and 2-(2-phenylethyl) chromone in agarwood met the basic requirements of the national standard for agarwood grading at 12 months. This study reports for the first time that *Fusarium equiseti* promotes agarwood formation in *A. sinensis*. Importantly, during our experimental period (up to 12 months post-inoculation), the trees inoculated with *F. equiseti* showed no signs of decay or mortality attributable to the fungal treatment, indicating its potential suitability under these conditions. It should be noted that the primary objective of this work was to screen for effective inducing fungi, and we did not conduct long-term studies specifically designed to assess the pathogenic potential or decay-causing capabilities of various fungi on mature agarwood trees over multiple years.

Most endophytes synthesize bioactive compounds conferring plant pathogen defense, with some possessing pharmacological potential [33,34]. *Fusarium* spp., common endophytes, produce terpenoids, polyketides, nonribosomal peptides, and anthraquinones exhibiting antibacterial, anti-inflammatory, and cytotoxic activities [35,36,37,38]. Although *Fusarium equiseti* is a known pathogen causing crop decay in *Averrhoa carambola* [39], *Actinidia chinensis* [40], and *Astragalus membranaceus* [41], studies report its antibacterial properties [42], disease suppression [43], plant growth promotion, and L-asparaginase production (an antitumor agent) in *Potentilla anserina* rhizosphere [44]. These functional traits may underlie the agarwood induction mechanism in *A. sinensis*, warranting further investigation.

Macroscopic and anatomical analyses revealed characteristic agarwood features in induced *A. sinensis*. The wood exhibits a diffuse-porous structure with large-diameter thin-walled vessels, inconspicuous axial parenchyma, and abundant inner phloem. A key macroscopic indicator of fungal induction efficacy is the extent of xylem discoloration [45]. Agarwood deposition occurs exclusively in phloem and wood ray parenchyma following external injury or stress, manifesting as dark brown tissue with a distinct aroma [46]. This process correlates with impaired vessel transport function [47]. When damaged by external environmental factors, starch degradation initiates [48], organic transport is disrupted, and wound repair triggers agarwood deposition. The weakly acidic, humid wood interior with rich carbohydrates facilitates fungal colonization, accelerating resin accumulation [49,50]. In this study, fungal inoculation induced progressive expansion and darkening of xylem discoloration around inoculation sites over 12 months. *F. equiseti* parasitizes *A. sinensis* xylem, provoking host defense responses that generate oleoresin-containing agarwood. The differential xylem colonization areas observed among fungi under identical conditions suggest variable infectivity, supporting xylem discoloration extent as a potential proxy for evaluating fungal induction efficacy in artificial inoculation systems.

Chemical analysis confirmed the efficacy of *F. equiseti* induction. Sesquiterpenes, key aromatic and bioactive constituents of agarwood with anticancer and neuropharmacological potential [12], increased progressively over time, consistent with trends observed in chemically [51] and fungally [52] induced *Aquilaria* species. Concurrent rises in aromatic hydrocarbons and aldehydes likely contribute to odor development. Crucially, *F. equiseti*-treated samples exhibited significantly higher agarotetrol accumulation across all timepoints compared to other methods. Furthermore, ethanol extraction (a standard resin quantifier [53]) yielded 17.69% after only 12 months, surpassing values from mixed-liquid induction (13.61% at 20 months) [54] and fire-induced methods (7.49% at 24 months) [55]. Although it does not match wild agarwood, this yield meets medicinal standards with markedly shorter induction time, lower cost, and stable efficacy. These results demonstrate that *F. equiseti* induces stable and effective agarwood formation. We propose that extended induction periods hold potential for yielding higher-quality resin, offering a sustainable and scalable production alternative for *A. sinensis* that enhances yield while conserving natural resources.

## Figures and Tables

**Table 1:** HPLC elution conditions.

Time (min)	Mobile Phase A (%)	Mobile Phase B (%)
0~10	15→20	85→80
10~19	20→23	80→77
19~21	23→33	77→67
21~39	33	67
39~40	33→35	67→65
40~50	35	65
50.1~60	95	5

**Table 2:** HPLC elution conditions.

Time (min)	Mobile Phase A (%)	Mobile Phase B (%)
0~7	10→20	90→80
7~13	20→25	80→75
13~24	25→30	75→70
24~40	30→35	70→65
40~41	35	65
41~58	35→42	65→58
58~82	42→60	58→40
82.1~92	95	5

**Table 3:** GC-MS compound summary table of agarwood samples.

No.	Compound	Rel.% (2 m)	Rel.% (4 m)	Exp. RI (2 m)	Exp. RI (4 m)	Ref. Corr.	Source (Ramp Rate)
1	Ethanol	0.27	3.31	447	441	440	NIST EO_ALKENE (5 °C/min)
2	Acetic acid	0.94	5.93	590	582	587	NIST TERPENOIDS (5 °C/min)
3	1-Methoxy-2-propanol	0.33	2.56	648	642	650	NIST EO_ALKENE (5 °C/min)
4	Heptane	0.17	-	701	-	700	Alkanes Theory (C7)
5	2-Ethyl-3-methylbutanal	0.05	0.66	752	752	750	NIST EO_ALKENE (5 °C/min)
6	2-Methylpentanal	0.1	-	889	-	902	Adams (2007, 3 °C/min)
7	3-MethylButanal	0.13	1.48	910	905	911	Adams (2007, 3 °C/min)
8	Benzaldehyde	0.22	0.1	950	950	951	Adams (2007, 3 °C/min)
9	2-Pentylfuran	0.08	0.29	980	983	981	Adams (2007, 3 °C/min)
10	Hexanal	0.59	2.05	1066	1083	1071	Adams (2007, 3 °C/min)
11	Nonanal	1.86	1.65	1095	1095	1091	Adams (2007, 3 °C/min)
12	Naphthalene	0.14	0.61	1172	1180	1171	Adams (2007, 3 °C/min)
13	Heptanal	-	0.22	-	1175	1171	Adams (2007, 3 °C/min)
14	1-Methylnaphthalene	0.03	-	1184	-	1181	Adams (2007, 3 °C/min)
15	Decanal	0.21	0.17	1198	1202	1196	Adams (2007, 3 °C/min)
16	Phenol	0.43	-	1218	-	1220	NIST EO_ALKENE (5 °C/min)
17	1-Pentanol	0.13	0.89	1240	1238	1241	Adams (2007, 3 °C/min)
18	3-Methyltridecane	0.05	-	1275	-	1276	Adams (2007, 3 °C/min)
19	Octanal	0.17	0.51	1285	1288	1279	Adams (2007, 3 °C/min)
20	1-Octanol	0.1	-	1290	-	1291	Adams (2007, 3 °C/min)
21	Tridecane	0.06	0.22	1300	1301	1300	Alkanes Theory (C13)
22	β-Elemene	0.38	-	1320	-	1329	Adams (2007, 3 °C/min)
23	1,6-Dimethylnaphthalene	0.09	-	1350	-	1351	Adams (2007, 3 °C/min)
24	3-Methyltetradecane	0.73	-	1375	-	1376	Adams (2007, 3 °C/min)
25	4-Methyltetradecane	0.6	-	1380	-	1381	Adams (2007, 3 °C/min)
26	Tetradecane	0.76	0.85	1400	1400	1400	Alkanes Theory (C14)
27	5-Methyltetradecane	0.49	-	1410	-	1411	Adams (2007, 3 °C/min)
28	3-Methyl-5-propylnonane	-	0.67	-	1438	1441	Adams (2007, 3 °C/min)
29	alloaromadendrene	0.38	0.46	1453	1460	1451	Adams (2007, 3 °C/min)
30	1-Heptanol	0.03	-	1455	-	1461	Adams (2007, 3 °C/min)
31	2-Methylpentadecane	1.06	0.54	1475	1476	1476	Adams (2007, 3 °C/min)
32	2,6,10-Trimethyldodecane	0.2	-	1480	-	1481	Adams (2007, 3 °C/min)
33	4-Methylpentadecane	1.11	-	1480	-	1481	Adams (2007, 3 °C/min)
34	2-Ethyl-1-hexanol	0.41	0.9	1480	1482	1481	Adams (2007, 3 °C/min)
35	Pentadecane	3.02	1.82	1500	1502	1500	Alkanes Theory (C15)
36	7-Methylpentadecane	1.29	0.75	1502	1502	1501	Adams (2007, 3 °C/min)
37	2,6,10-Trimethyltridecane	1.5	0.9	1588	1591	1590	Adams (2007, 3 °C/min)
38	Hexadecane	3.28	1.67	1600	1601	1600	Alkanes Theory (C16)
39	β-Eudesmol	1.98	3.83	1645	1652	1640	Adams (2007, 3 °C/min)
40	2,6,10,14-Tetramethylpentadecane	1.77	0.62	1858	1862	1861	Adams (2007, 3 °C/min)

Note: Compounds are listed in order of elution from a DB-5MS capillary column. Rel.% (2 m/4 m): Relative percentage content at 2/4 months post-inoculation; Exp. RI (2 m/4 m): Experimental retention indices calculated from C_8_–C_40_ n-alkanes (Kovats system); Ref. Corr.: Reference RI values corrected for 5 °C/min ramp rate [Ref. Corr. = RI lit − 4.5 × (5 − RampRate lit)]; Source: Data sources (Adams, 2007 [22]; Van den Dool & Kratz (Alkanes Theory), 1963 [23]; NIST 2020 [24]).

**Table 4:** Core-associated compounds.

No.	Compound	2 m (%)	4 m (%)	Metabolic Function
39	β-Eudesmol	1.98	3.83	Characteristic aroma marker of agarwood; induces resin synthesis
2	Acetic acid	0.94	5.93	Wound-response signaling molecule; promotes sesquiterpene cyclization
22	β-Elemene	0.38	-	Early biomarker of fungal stress
9	2-Pentylfuran	0.08	0.29	Indicator of xylem oxidative damage; positively correlates with agarwood color formation

**Table 5:** Secondary Associated Compounds.

No.	Compound	2 m (%)	4 m (%)	Mechanism
10	Hexanal	0.59	2.05	Lipid peroxidation product; activates defense gene expression
1	Ethanol	0.27	3.31	Anaerobic metabolism marker; promotes resin exudation
8	Benzaldehyde	0.22	0.1	Phenylalanine pathway metabolite; precursor of agarwood’s “sweet aroma”
35	Pentadecane	3.02	1.82	Degradation product of the cuticular wax layer; reflects physical trauma extent
38	Hexadecane	3.28	1.67	Synergizes with pentadecane in indicating wound response
29	alloaromadendrene	0.38	0.46	Byproduct of sesquiterpene synthesis

**Table 6:** Agarotetraol content.

No.	Treatments	Content of Agarotetrol (%)		
		Two Months	Four Months	Six Months
1	YB-1	0.034 ± 0.015 ^b^	0.039 ± 0.012 ^b^	0.038 ± 0.003 ^b^
2	PDA	0.014 ± 0.004 ^c^	0.025 ± 0.023 ^c^	0.004 ± 0.002 ^c^
3	Cold drill	0.006 ± 0.001 ^d^	0.002 ± 0.002 ^d^	0.037 ± 0.004 ^b^
4	Chinese Agarwood	0.173 ± 0.011 ^a^		

Note: Different lowercase letters in the same column indicate a significant difference at the *p* < 0.05 level.

**Table 7:** Content determination results.

Treatments	Ethanol Extractable Content (%)	The Sum of Relative Contents of 2-[2-(4-methoxyphenyl) ethyl] Chromone and 2-(2-phenylethyl) Chromone (%)
Cold drill	10.33 ± 0.46 ^a^	-
PDA	11.10 ± 0.57 ^a^	-
YB-1	17.69 ± 0.37 ^b^	2.13 ± 0.14 ^a^
Chinese Agarwood	28.71 ± 0.21 ^c^	5.42 ± 0.13 ^b^

Note: Different lowercase letters in the same column indicate a significant difference at the *p* < 0.05 level.

## Data Availability

The original contributions presented in this study are included in the article. Further inquiries can be directed to the corresponding author.
